# Possible use of Digital Variance Angiography in Liver Transarterial Chemoembolization: A Retrospective Observational Study

**DOI:** 10.1007/s00270-023-03420-2

**Published:** 2023-04-19

**Authors:** Pierleone Lucatelli, Bianca Rocco, Simone Ciaglia, Leonardo Teodoli, Renato Argirò, Boris Guiu, Luca Saba, Giulio Vallati, Stavros Spiliopoulos, Lorenzo Patrone, Marcell Gyánó, István Góg, Szabolcs Osváth, Krisztian Szigeti, János P. Kiss, Carlo Catalano

**Affiliations:** 1 University Hospital “Policlinico Umberto I, Sapienza University of Rome, Rome, Italy; 2 Department of Interventional Radiology, University of Rome Tor Vergata, Rome, Italy; 3 Montpellier University Hospital, Montpellier, France; 4 Department of Medical Sciences, University of Cagliari, Cagliari, Italy; 5 Interventional Radiology Unit of “IRCCS Istituto Nazionale Tumori Regina Elena”, Rome, Italy; 6 Department of Radiology, Interventional Radiology Unit, National and Kapodistrian University of Athens, Athens, Greece; 7 West London Vascular and Interventional Centre, Northwick Park Hospital, London, UK; 8 Department of Interventional Radiology, Heart and Vascular Center, Semmelweis University, Budapest, Hungary; 9 Kinepict Health Ltd, Budapest, Hungary; 10 Department of Vascular and Endovascular Surgery, Heart and Vascular Center, Semmelweis University, Budapest, Hungary; 11 Department of Biophysics and Radiation Biology, Semmelweis University, Budapest, Hungary

**Keywords:** Digital variance angiography (DVA), Digital subtraction angiography (DSA), Transarterial chemoembolization (TACE), Image quality (IQ), Contrast-to-noise ratio (CNR)

## Abstract

**Purpose:**

Digital variance angiography (DVA), a recently developed image processing technology, provided higher contrast-to-noise ratio (CNR) and better image quality (IQ) during lower limb interventions than digital subtraction angiography (DSA). Our aim was to investigate whether this quality improvement can be observed also during liver transarterial chemoembolization (TACE).

**Materials and Methods:**

We retrospectively compared the CNR and IQ parameters of DSA and DVA images from 25 patients (65% male, mean ± SD age: 67.5 ± 11.2 years) underwent TACE intervention at our institute. CNR was calculated on 50 images. IQ of every image set was evaluated by 5 experts using 4-grade Likert scales. Both single image evaluation and paired image comparison were performed in a blinded and randomized manner. The diagnostic value was evaluated based on the possibility to identify lesions and feeding arteries.

**Results:**

DVA provided significantly higher CNR (mean CNR_DVA_/CNR_DSA_ was 1.33). DVA images received significantly higher individual Likert score (mean ± SEM 3.34 ± 0,08 vs. 2.89 ± 0.11, Wilcoxon signed-rank *p* < 0.001) and proved to be superior also in paired comparisons (median comparison score 1.60 [IQR:2.40], one sample Wilcoxon *p* < 0.001 compared to equal quality level). DSA could not detect lesion and feeding artery in 28 and 36% of cases, and allowed clear detection only in 22% and 16%, respectively. In contrast, DVA failed only in 8 and 18% and clearly revealed lesions and feeding arteries in 32 and 26%, respectively.

**Conclusion:**

In our study, DVA provided higher quality images and better diagnostic insight than DSA; therefore, DVA could represent a useful tool in liver TACE interventions.

**Level of evidence:**

III Non-consecutive study**.**

## Introduction

Transarterial chemoembolization (TACE) represents the standard of care for early or intermediate stage liver cancer [1] and hepatic metastases from colorectal cancer in patients not suitable for surgery/ablation [2]. The updated guideline documents clearly indicate the need of advanced imaging modality to guide such interventions, thus allowing a better clinical response. Patients selected for TACE, may need multiple sessions of treatment, depending on the total tumour burden. In this respect, radiation exposure and contrast media administration throughout the procedure should be kept at the minimum [3].

Digital variance angiography (DVA), a recently developed image processing technology, might address these problems. The method is based on the principles of kinetic imaging [4]. In contrast with digital subtraction angiography (DSA), DVA does not use a mask image, but calculates standard deviation for each pixel in an unsubtracted image series. This statistical analysis enhances the contrast agent-generated signal and suppresses the noise, therefore provides a higher contrast-to-noise ratio (CNR) and an improved image quality (IQ). This excess quality, also termed as quality reserve, has already been demonstrated in lower limb angiography using either iodinated contrast media (ICM) [5–7] or carbon dioxide (CO2) [8, 9] as a contrast agent. The quality reserve of DVA provides opportunity for dose management solutions [10, 11], which could be beneficial not only in lower limb procedures but also, and even more, in a wide range of endovascular interventions, particularly the ones involving visceral vessels which usually require higher radiation exposure to the patient and the operator.

The aim of the present study is to compare the performance of DSA and DVA in liver TACE procedures. For this reason, we compared the CNR and IQ of the two image processing technologies, and also their specific diagnostic value to identify and characterise liver tumours and feeding arteries of lesions in patients with hepatocellular cancer.

## Materials and Methods

In our single-centre observational study, angiographical image series of 25 patients affected by hepatocarcinoma and who underwent TACE, were retrospectively collected and processed. All procedures were in accordance with the ethical standards of the institutional and/or national research committee and with the 1964 Helsinki Declaration and its later amendments. Because of the retrospective nature of the study, informed consent was not required. All indications for TACE treatment were decided by a multidisciplinary tumour board (composed by a surgeon, oncologist, hepatologist and body radiology).

## Study Design

Two pre-embolization acquisitions (a frontal and an oblique view) were included in the study from each patient. The same unsubtracted series was used to generate DSA and DVA images using the Siemens Syngo and the Kinepict Medical Imaging Tool software, respectively. The CNR values and the ratio were calculated for each image pair, and the IQ was evaluated by 4-grade Likert scales in blinded and randomized surveys. The visual evaluation included single image scoring and paired image comparison (for details see below). The diagnostic value was evaluated by the ability of the readers to identify lesions and their feeding arteries.

## Image Acquisition

TACE procedures were performed by two interventional radiologists with more than 13 years of experience according to the standardized institutional protocol. Following femoral or radial access under local anaesthesia, a diagnostic catheter (Simmons 1, Cordis, Hialeah, FL, USA) was introduced in the common hepatic artery, and two angiograms (an anteroposterior and 25° right anterior oblique) were acquired at 3 FPS on a Siemens Artis Zee system and a Syngo XWP VB21N workstation (Siemens Healthcare). A Medrad Avanta Mark V ProVis automatized injector (Bayer) was used for injecting 12–15 ml/injection contrast media (Ultravist 370, Bayer) at 3–5 ml/s flowrate from a 4 Fr catheter positioned in proper hepatic artery. A cone-beam CT was also acquired to obtain the liver tumour vascularization map and lesion’s feeders detection. On the basis of these two imaging modalities, the best location to perform embolization by microcatheter (Progreat, Terumo, Tokyo, Japan) was identified, and the embolization was occurred using LifePearl (Terumo, Japan) 100 µm or DC beads M1 (Boston Scientific) microspheres.

## Image Processing

Stacked DSA images were generated using the opacification function, and the brightness/contrast was optimised on the Syngo XWP VB21N workstation (Siemens Healthineers AG, Erlangen, Germany). The raw unsubtracted acquisitions were exported from the Siemens workstation, and the corresponding DVA images were generated and post-processed retrospectively from the same unsubtracted raw series using the Kinepict Medical Imaging Tool v.5.0 (Kinepict Health, Budapest, Hungary). The post-processed DSA and DVA images were saved in DICOM format and were used for CNR calculations and visual evaluation.

## CNR Analysis

For CNR measurements, regions of interest (ROI) were defined on vessels and background regions by using Image J (v.2.0.0-rc-68/1.52e, Creative Common License, NIH) Rueden [12]. The vascular and adjacent background ROI were placed in pairs. The same ROI sets were used on all corresponding DSA and DVA images. ROI positions were adjusted when patient positioning or pixel shifting caused slight geometric differences. CNR values were calculated for all ROI pairs individually according to the following formula, wherein ${Mean}_{v}$ and ${Mean}_{b}$ referred to mean pixel intensity values of the vascular and background ROI, respectively, and ${Std}_{b}$ being the background standard deviation (Rose) [13]

$${\text{CNR}} = \frac{{\left| {{\text{Mean}}_{v} - {\text{Mean}}_{b} } \right|}}{{{\text{Std}}_{b} }}$$

CNR_DVA_/CNR_DSA_ ratios (*R*) for each corresponding DVA and DSA ROIs were calculated.

## Visual Evaluation

Visual evaluation was performed by five interventional radiologist experts in the field of liver catheter-based treatments with at least 15 years of experience. The readers were not involved in the treatment of the enrolled patients.

In the single image evaluation only one, randomly selected DSA or DVA image was visible at a time. The readers, blinded to the processing modality, evaluated the IQ using the following 4-grade Likert scale:

1. Poor IQ, vascular structures are not distinguishable
2. Low IQ, good visualization of lobar vessels only
3. Medium IQ, good visualization of lobar and segmental vessels
4. Good IQ, good visualization of lobar, segmental and subsegmental vessels

The diagnostic value was evaluated in this survey as the readers had to judge the visibility of lesions and feeding arteries using the following options:

1. Not visible
2. Suspected but not definitive
3. Clear identification

The IQ and diagnostic value were also evaluated in a paired comparison, when a DSA and the corresponding DVA image were shown simultaneously (but the image type was undisclosed). The readers had to select a preferred image and compare the IQ and diagnostic value based on the visibility of small vessels, lesions and feeding arteries. The following 5-grade preference scale was used:

1. No difference
2. Slightly better
3. Moderate differences
4. Major differences
5. Better in every aspect

The image type was never disclosed, and the order of image pairs (i.e. the appearance on the left or right side of the screen) or the appearance of single images was randomized. Thus, all rating scales were implemented in blinded and randomized web-based surveys, and the data were collected automatically in a database for later processing.

## Statistical Analysis

Calculations of CNR and *R* means, medians and interquartile ranges were performed using Excel 2016 (Microsoft, Redmond, WA). CNR values were compared by the Wilcoxon signed-rank test (Prism 8.4.2., GraphPad).

For visual evaluation scores, the mean and standard error of mean (SEM) were calculated. Because of the non-Gaussian distribution of data, the median and interquartile range (Q1–Q3) were also determined. The single image scores were compared by the Wilcoxon signed-rank test, the results of the paired image comparison were analysed by the one sample Wilcoxon test to investigate the relation of DSA and DVA images (equal quality or superiority), whereas the single image diagnostic results were analysed by the two-sided *Z* test. Kendall’s *W* was calculated to describe interrater agreement. The level of significance was set at p < 0.05 in all tests.

## RESULTS

### Patients

Patients (*n* = 25, 65% male, mean ± SD age: 67.5 ± 11.2 years) with previously diagnosed hepatocarcinoma nodules received TACE treatment between January 2021 and June 2021 at the University Hospital  'Policlinico Umberto I', and were retrospectively enrolled for image analysis in a consecutive manner.

## CNR Results

**Fig. 1:**
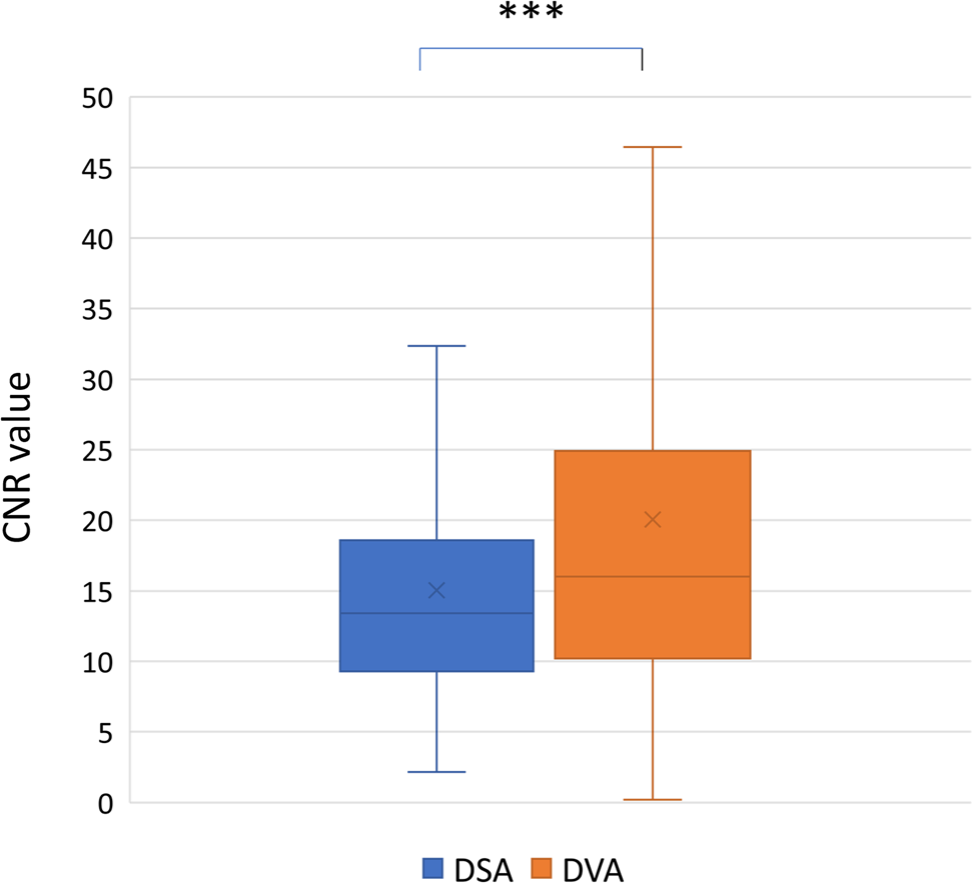
Contrast-to-noise ratio (CNR) results. The box and whisker plots show the median (line), mean (x), interquartile range (box) and internal fences (whiskers) of CNR values in each group. Data sets were analysed by the Wilcoxon signed-rank test (***p < 0.001). DVA: digital variance angiography and DSA: digital subtraction angiography

A total of 686 ROI pairs were measured on 50 images. Table 1 summarizes the results of the CNR measurements. The mean (± SEM) CNR of DVA images (20.14 ± 0.58) was significantly higher than that of DSA images (15.02 ± 0.31, Wilcoxon signed-rank *p* < 0.001), the R (CNR_DVA_/CNR_DSA_) value was 1.34 ± 0.04 (Fig. 1).

**Table 1:** Contrast-to-noise ratio (CNR) analysis. Data are expressed as mean ± standard error of mean (SEM), and as median and interquartile range (IQR). Wilcoxon signed-rank test was used for statistical comparison, significance level was set at *p* < 0.05. DVA: digital variance angiography and DSA: digital subtraction angiography

	CNR		R	Wilcoxon signed-rank test
	DSA	DVA	DVA/DSA	DSA vs. DVA
Mean ± SEM	15.01 ± 0.30	20.14 ± 0.58	1.34 ± 0.02	*p* < 0.001
Median (IQR)	13.34 (9.21)	16.02 (14.89)	1.24 (0.69)

## Visual Evaluations Results

**Fig. 2:**
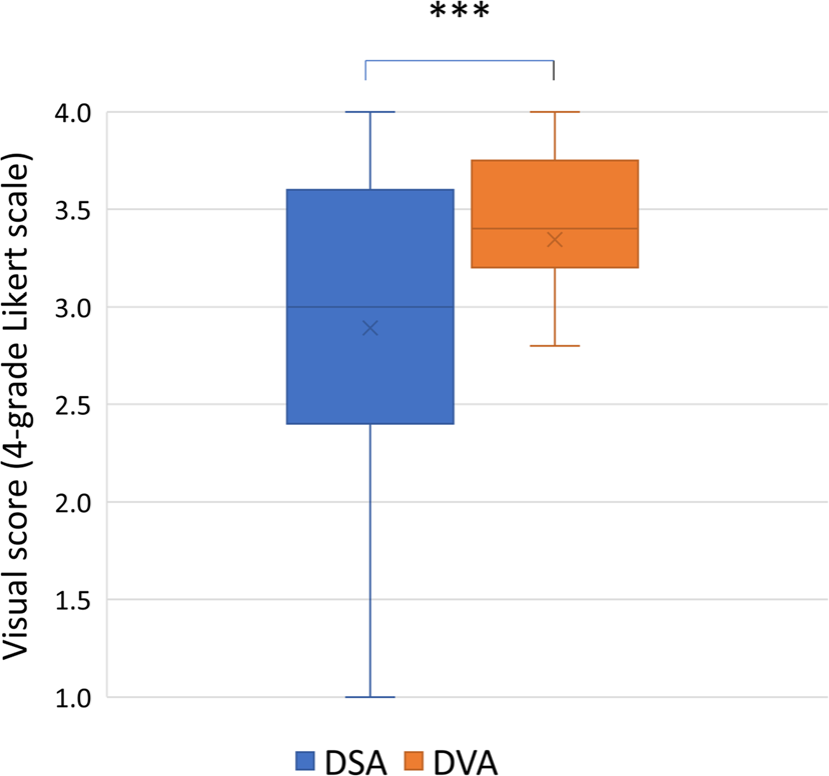
Single image evaluation results. A 4-grade Likert score was used in the blinded, randomized survey (see Materials and Methods) to evaluate the image quality of DSA and DVA images. The box and whisker plots show the median (line), mean (x), interquartile range (box) and internal fences (whiskers) of single image scores values in each group. Data sets were analysed by the Wilcoxon signed-rank test (***p < 0.001). DVA: digital variance angiography and DSA: digital subtraction angiography

Readers evaluated 50 DSA and 50 DVA images in blinded, randomized manner. In the single image evaluation (when only one image appeared on the screen), DVA images received significantly higher Likert score (mean ± SEM DVA 3.34 ± 0,08 vs. DSA 2.89 ± 0.11, Wilcoxon signed-rank *p* < 0.001), and similar difference was seen in the median and IQR values (DSA 3.0, IQR 1.2 vs. DVA 3.4, IQR 0.55) (Fig. 2). The Kendall W values showed substantial agreement for DSA (0.610, *p* < 0.001) and moderate agreement for DVA (0.423 *p* < 0.001).

**Fig. 3:**
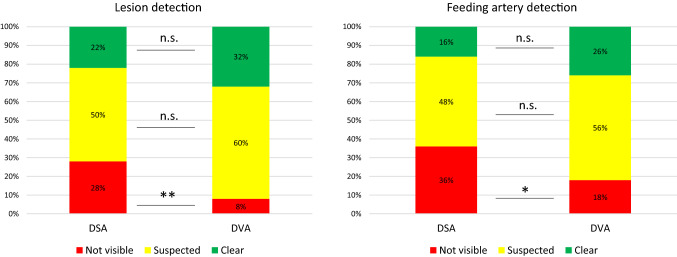
Comparison of the diagnostic value of DSA and DVA. Readers classified each image based on the ability to identify structures (lesion and feeding arteries) being critically important in TACE interventions. Two-sided Z test was used for the statistical comparison of percentage data (*p < 0.05, **p < 0.01). TACE: TransArterial ChemoEmbolization; DSA: digital subtraction angiography and DVA: digital variance angiography

The diagnostic value was also evaluated during the single image survey. Readers evaluated the visibility of lesions and feeding arteries. DVA failed to visualize these critically important structures in significantly less images than DSA (8 vs. 28% for lesions, two-sided Z *p* < 0.01; and 16 vs. 32% for feeding arteries, *p* < 0.05). There was no significant difference in the proportion of suspected and clearly visualized structures, although DVA showed a tendency to enhance visualization, as it increased by 45 and 63% the number of images with clear lesion and feeding artery identification, respectively (Fig. 3). The interrater agreement was moderate for both feeding artery (Kendall’s W value: DSA 0.541, *p* < 0.001, DVA 0.551, *p* < 0.001) and lesion detection (Kendall’s W value: DSA 0.564, *p* < 0.001, DVA 0.561, *p* < 0.001).

**Fig. 4:**
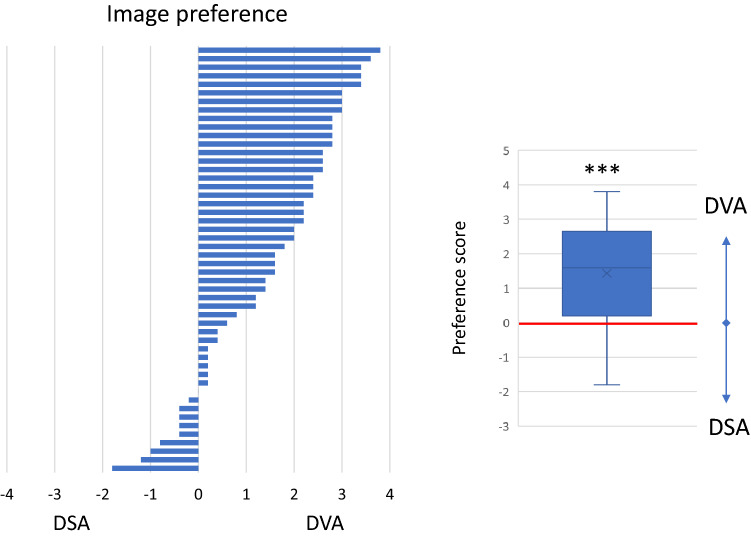
Paired comparison of DSA and DVA images. Readers compared the image quality and diagnostic value of image pairs in a blinded, randomized manner, and expressed their image preference using a 5-grade preference scale. The left panel shows the distribution of the average preference scores of individual image pairs. The box and whiskers plot shows the mean (x), median (line), interquartile range (box) and internal fences (whiskers) of the complete image set. The 0 line represents the theoretical equal quality level. Data were analysed by the one-sample Wilcoxon test (***p < 0.001). DSA: digital subtraction angiography and DVA: digital variance angiography

**Fig. 5:**
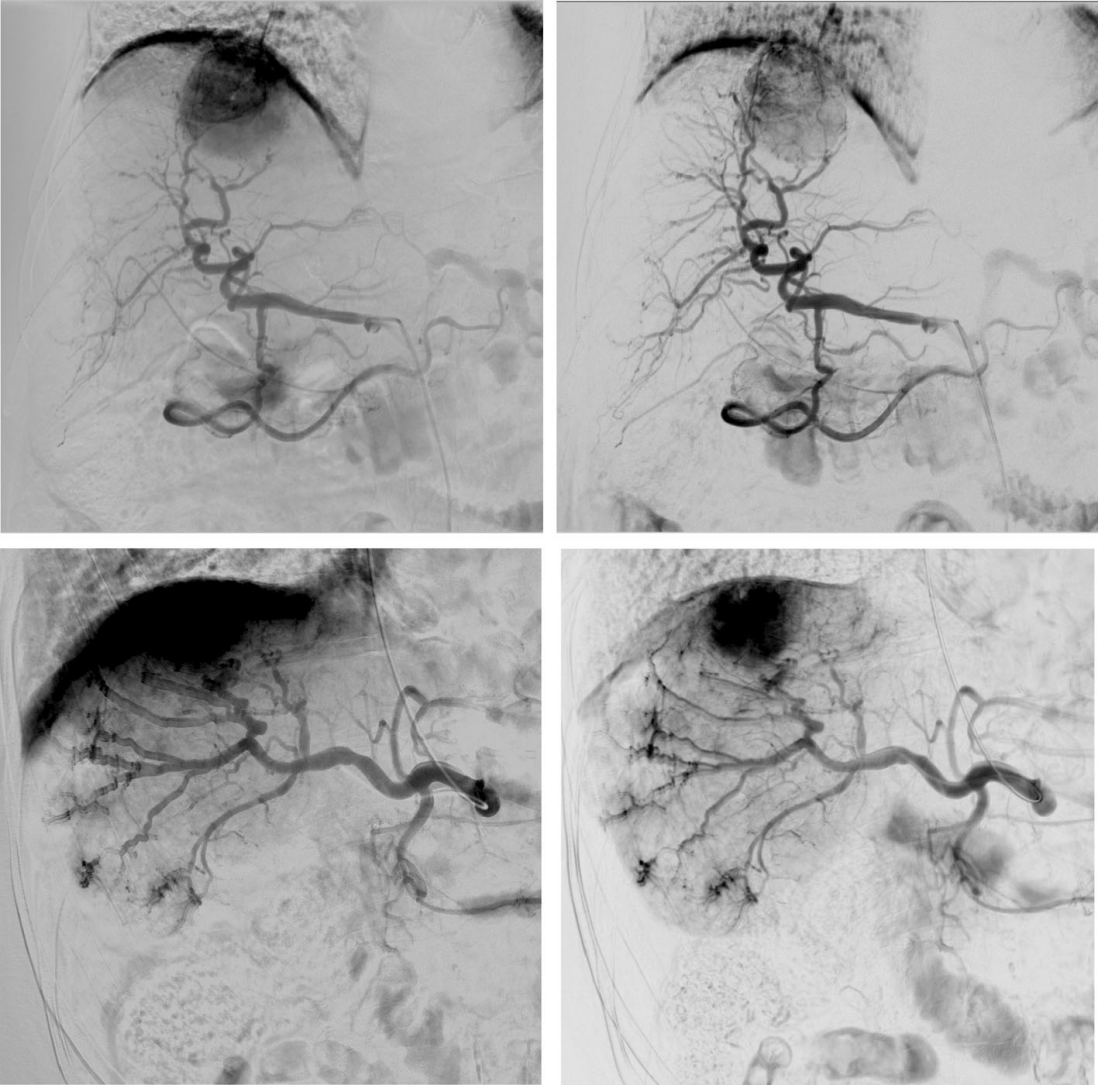
Comparison of a representative DSA (left panels) and DVA (right panels) image pairs. More millimetre to submillimetre arterial vessels can be depicted on the DVA images, and both the lesions and the feeding arteries can be evaluated more clearly. The DSA and DVA images were generated from the same unsubtracted image series using the Siemens Syngo or the Kinepict Medical Imaging Tool software, respectively

The paired comparison allowed a side-by-side evaluation of DSA and DVA images in a blinded and randomized manner. DVA was the preferred image in 80% of comparisons (Fig. 4, left panel), and the average score (mean ± SEM) of the whole image set was 1.44 ± 0.21, the median score was 1.60 (IQR 2.4), significantly different from 0 (one-sample Wilcoxon *p* < 0.001), which represented the equal quality level, indicating the superiority of DVA images (Fig. 4, right panel). The interrater agreement was also significant (Kendall’s W value: 0.575, *p* < 0.001). Representative image pairs are shown on Fig. 5.

## Discussion

The major aim of our study was to compare the performance of DSA and DVA in liver TACE intervention. Although the quality reserve of DVA has already been demonstrated in lower limb [5–8] and carotid [10] angiography, these anatomical regions are very different from the abdominal area, where bone shadow is less emphasized but bowel gas and intestinal movement artefacts might be significant. We have analysed the CNR, an objective predictor, and visual evaluation, a subjective descriptor of image quality. In addition, the diagnostic value of the image types was also assessed.

In our study, CNR was significantly higher in DVA than DSA imaging. In terms of absolute values, the CNR DVA values were lower than those reported in other DVA applications: in lower extremity regions [5–8] and endovascular carotid interventions [10]. This may be due to several factors that may have influenced the final results. During TACE procedures, motion artefacts may occur due to breathing, cardiac pulsations, and bowel gas may cause a loss of important information [14]. Regardless the fact that the obtained absolute CNR value was lower than in other anatomical regions, this was sufficient to provide an advantage of DVA technology over standard DSA in visual and diagnostic evaluation. Further DVA development, currently under investigation, may compensate movement artefact and eliminate bowel gases, thus potentially further ameliorating diagnostic performance even in the liver field of application.

The visual evaluation results showed that DVA provides higher image quality than DSA (Fig. 2), even if the interrater agreement was higher for the DSA images (probably, because this is the usual image type, the readers met before). Due to this quality advantage, DVA was able to improve the percentage of visible lesions (DVA 32% vs. DSA 22%) (Fig. 3) and to reduce the number of cases, in which angiography did not depict any lesion (DVA 8% vs. DSA 28%). Moreover, in terms of feeder vessel detection, readers evaluation also revealed a significant advantage of DVA technology over standard DSA. The blinded comparison of corresponding DVA and DSA images shows even more evidently the superiority of DVA in terms of overall diagnostic value. These data clearly demonstrate, how DVA technology may provide a major clinical benefit by allowing the operator to see more lesions and to better identify feeding vasculature during liver embolization procedure. DVA may be valuable also during more challenging TACE interventions (e.g. in case of hypo-vascular lesions or for tumours located high in the dome), when the identification and characterization of lesions by standard imaging is more difficult, and consequently, their endovascular treatment is more complicated.

Our results might have further clinical implications. The observed quality reserve of DVA might be used to reduce the radiation exposure to patients and operators, and the amount of contrast medium administered, as already demonstrated earlier in other clinical settings: This technology allowed 50% reduction of contrast media in carotid angiography [10] and 70% reduction of radiation dose in lower limb angiography [11] without compromising the image quality and diagnostic value of stationary acquisitions. The possibility to reduce contrast medium and/or radiation dose administered during TACE interventions might be especially important, if we consider that patients selected for transarterial intervention may have impaired renal function and usually need more than one treatment session. Obviously, validation of these claims requires further clinical trials, but this study provides a rationale for the initiation of prospective dose management trials in TACE.

The study has some limitations. The number of patients is relatively low, in line with the design of a small cohort proof-of-concept study, even though the number of evaluated images fully complies with the recommendations of an FDA guideline on the testing X-ray imaging devices [15]. The breathing artefacts and bowel gases obviously impair the performance of DVA, which was reflected also by the smaller difference in CNR values between DSA and DVA. The quality reserve of DVA is clearly demonstrated even under the current conditions, and we are confident that new compensatory algorithms currently under development (which will reduce or eliminate these disturbing factors) will increase even more the gap between the two diagnostic modalities. Another possible limitation of this study is that the image processing was done off-line in a retrospective manner as the raw data were exported from the angiography computer. Nevertheless, the technology can be operated in quasi real-time, as the transfer and data processing take usually less than 2 s. Thus, the DVA image appears on the operating room monitor almost immediately. Of course, due to these circumstances, the technology cannot be used in fluoroscopy, but otherwise the 1–2 s delay is tolerable for stationary acquisitions. The advantages of DVA in real-time operation has already been validated in a previous study on CO_2_-assisted lower limb interventions [9], but the possible benefits in TACE interventions should be investigated in the future in a live setting, when the technology is fully integrated with the angiography system and DVA images are available in real time in the operating room.

## Conclusion

The results of this study present the application of digital variance angiography (DVA) in liver embolization procedures. Our data indicate that DVA provides better image quality and more diagnostic information than DSA; therefore, it might be a useful new tool in TACE procedures for the treatment of liver tumours. The observed quality reserve of DVA might be used for radiation dose and contrast agent reduction in TACE; however, these claims need validation by further prospective clinical studies.
